# Combining Vascularization Strategies in Tissue Engineering: The Faster Road to Success?

**DOI:** 10.3389/fbioe.2020.592095

**Published:** 2020-12-08

**Authors:** Thomas Später, Emmanuel Ampofo, Michael D. Menger, Matthias W. Laschke

**Affiliations:** Institute for Clinical and Experimental Surgery, Saarland University, Homburg, Germany

**Keywords:** vascularization, microvascular fragments, arteriovenous loop, tissue engineering, blood vessels

## Introduction

Tissue engineering is an interdisciplinary field of biomedical research that aims for the restoration of tissue defects or even the replacement of complete organs (Griffith and Naughton, 2002). For this purpose, tissue constructs are generated by seeding stem cells or tissue-specific cells on three-dimensional biomaterials, also referred to as scaffolds. These materials should mimic the natural extracellular matrix to ideally support the physiological function and regenerative capacity of the seeded cells (Hutmacher et al., 2004; Hutmacher and Cool, 2007). Moreover, they should rapidly vascularize to guarantee sufficient oxygen supply and, hence, cellular survival after their implantation into a tissue defect (Blinder et al., 2015; Cerino et al., 2017). In fact, the lack of an adequate vascularization is a major reason for the failure of particularly extensive and complex materials for the treatment of large-scale tissue defects (Maggi et al., 2003; Kneser et al., 2006; Leibig et al., 2016; Weigand et al., 2018; Yuan et al., 2018).

**Figure 1:**
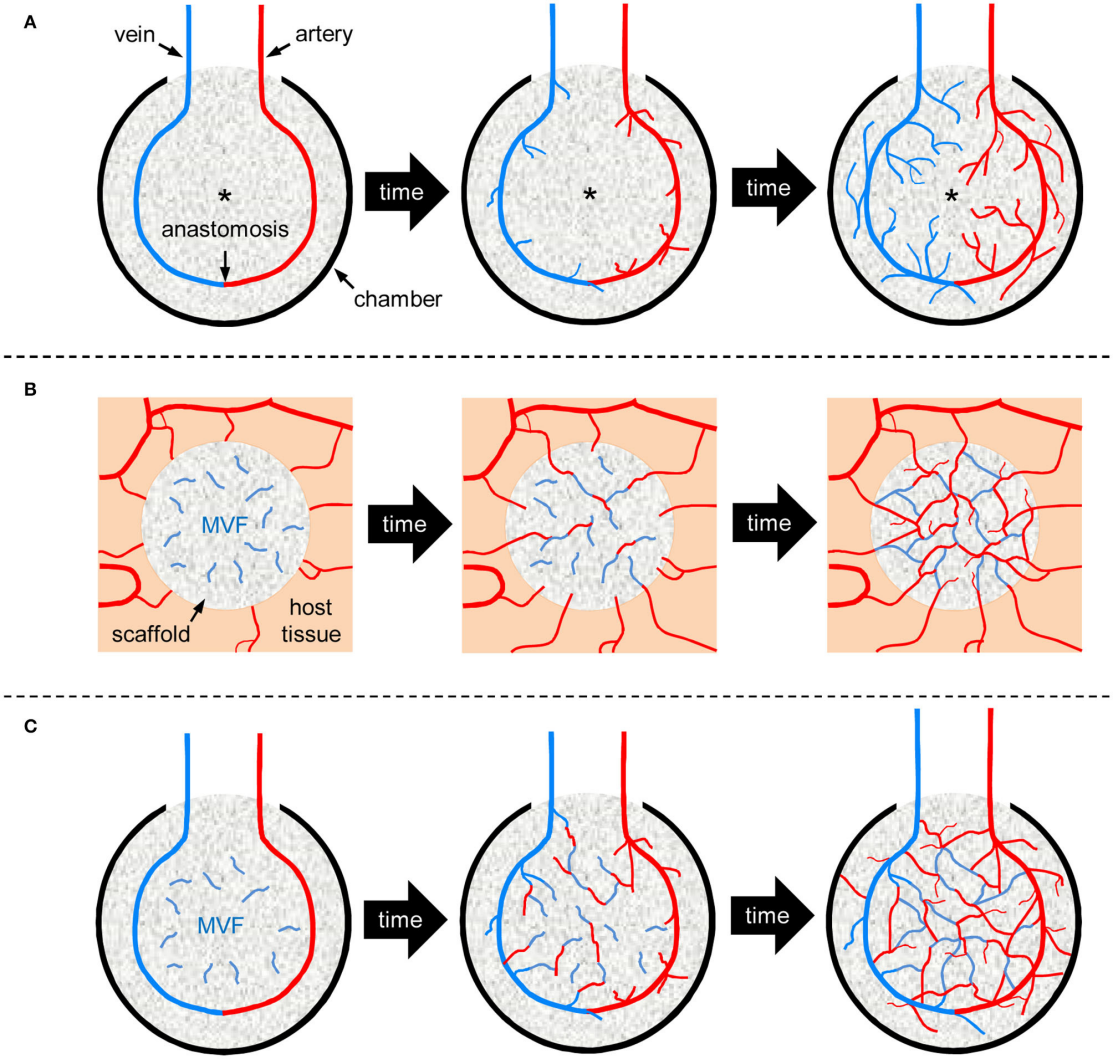
Schematic illustration of vascularization strategies for tissue engineering. **(A)** *AV loop-based vascularization:* An AV loop, i.e., an anastomosed artery and vein, is placed in a protecting scaffold-containing chamber. Shear stress-induced angiogenic sprouting out of the AV loop progressively vascularizes the surrounding scaffold over time. Due to the slow growth rate of new blood vessels, the center zone of the chamber (asterisk) suffers from hypoxia during the early phase of implantation. **(B)** *MVF-based vascularization:* A scaffold is seeded with MVF and implanted into a host tissue. The seeded MVF rapidly develop interconnections with each other and the blood vessels of the surrounding host microvasculature, leading to an early onset of blood perfusion within the entire implant. **(C)** *Combination of AV loop- and MVF-based vascularization:* An AV loop-containing chamber is additionally seeded with MVF. The seeded MVF rapidly interconnect with each other as well as with the microvessels growing out of the AV loop, resulting in a markedly accelerated vascularization over time.

A promising strategy to overcome this problem is the generation of pre-vascularized tissue by means of an arteriovenous (AV) loop. This approach has not only demonstrated promising results in various experimental studies (Lokmic et al., 2007, 2008; Beier et al., 2009; Arkudas et al., 2013; Weigand et al., 2018), but is also already applied in clinical practice (Wang and Chu, 1996; Asif et al., 2005; Laschke and Menger, 2016; Henn et al., 2019; Hernández-Enríquez et al., 2019; McEwan et al., 2019). The basic concept of this *in situ* strategy is the generation of an axially vascularized tissue using the patient's own body as a bioreactor (Reichenberger et al., 2010; Laschke and Menger, 2016; Radwan et al., 2018; Weigand et al., 2018). In detail, an anastomosis between an artery and vein results in an AV loop, which is subsequently transferred into an enclosed implantation chamber to provide an isolated *in vivo* environment (Figure 1A) (Mian et al., 2000; Lokmic et al., 2008). This chamber is either empty or contains a cell-free or cell-seeded scaffold that needs to be vascularized (Mian et al., 2000; Lokmic et al., 2008; Weigand et al., 2016). During the following time course, mechanical shear stress stimulates the angiogenic sprouting of new microvessels out of the AV loop, which ultimately leads to the filling of the chamber with fibrovascular tissue (Figure 1A) (Asano et al., 2005; Dong et al., 2012; Zhan et al., 2016). Once the tissue inside the chamber is fully vascularized, it can be removed together with the AV loop and transferred to a tissue defect of the same patient. The main advantage of this procedure is the fact that the generated tissue can be transplanted with its AV loop and fully developed vascular network and, thus, easily anastomosed to the local blood vessels at the final site of implantation (Weigand et al., 2018).

However, although multiple animal studies have demonstrated the successful formation of angiogenic sprouts within AV loop-containing chambers over time (Asano et al., 2005; Kneser et al., 2006; Dong et al., 2012), their complete vascularization may take up to 12 weeks (Mian et al., 2000). These findings indicate that AV loop-based vascularization is a rather time-consuming strategy, which therefore can be associated with a long hospitalization for patients.

To accelerate angiogenesis originating from AV loops, it has been suggested to use matrices as release systems for growth factors, such as vascular endothelial growth factor (VEGF) or basic fibroblast growth factor (bFGF) (Leibig et al., 2016). However, although growth factors stimulate angiogenic sprouting from pre-existing blood vessels, they can only accelerate the physiological growth rate of blood vessels, which is described to be only ~ 5 μm/h (Utzinger et al., 2015), to a certain extent. On the other hand, Matsuda et al. (2013) suggested the use of stem cells to promote angiogenesis within the tissue of AV loop-containing chambers. They found that, most likely due to the initial hypoxic conditions within the chambers, only a few stem cells survived and were successfully incorporated into the newly formed tissue (Matsuda et al., 2013). Hence, there is an urgent need for the establishment of more effective strategies to accelerate tissue vascularization around AV loops. To achieve this, we herein suggest the novel approach of combining AV loop-based vascularization with another effective vascularization strategy, i.e., the transplantation of adipose tissue-derived microvascular fragments (MVF).

## Mvf As Natural Vascularization Units

MVF represent a randomized mixture of biologically intact arteriolar, capillary and venular vessel segments, which can be rapidly isolated in large amounts from adipose tissue by means of mechanical dissection and enzymatic digestion (Frueh et al., 2017). They exhibit an intact vessel morphology with a central lumen, endothelial cells and stabilizing mural cells, such as pericytes (Laschke and Menger, 2015). Hence, they reassemble into new microvascular networks much faster than single cells and also develop interconnections to the surrounding host tissue after transplantation (Figure 1B) (Später et al., 2018). Moreover, their length of up to 150 μm allows them to bridge relatively wide distances within seeded matrices (Später et al., 2017a). Finally, MVF also secrete various pro-angiogenic growth factors and are a rich source of mesenchymal stem cells and endothelial progenitor cells (McDaniel et al., 2014; Laschke and Menger, 2016). Accordingly, MVF have already been shown to be effective natural vascularization units for random pattern-flaps (Nakano et al., 1998), superficial myocardium (Nakano et al., 1999), epicardial patches (Shepherd et al., 2007), volumetric muscle defects (Pilia et al., 2014), pancreatic encapsulating devices (Hiscox et al., 2008) as well as scaffolds for bone and skin tissue engineering (Laschke et al., 2012; Später et al., 2017b, 2020).

Based on these findings, it is obvious that MVF may also significantly accelerate the vascularization within an AV loop-containing chamber. It may be speculated that, under future clinical conditions, MVF are rapidly isolated from liposuctioned fat of patients and seeded on an appropriate scaffold during the time of surgical AV loop creation. Both components could then be transferred in the AV loop chamber in an intra-operative one-step procedure. During the following course, the seeded MVF would rapidly interconnect with each other as well as with the microvessels growing out of the AV loop (Figure 1C). This would markedly reduce the time required for a sufficient vascularization of the entire chamber.

## Proof of Concept and Clinical Translation

To analyze this approach under experimental conditions, we suggest to first perform animal studies in rats. In comparison to mice, the blood vessels in rats are larger and, thus, more suitable for anastomoses during the creation of AV loops. For this reason, the AV loop-technique has originally been established in rats (Korber and Flye, 1987) and since then been further developed in this species (Tanaka et al., 2000; Kneser et al., 2006; Arkudas et al., 2013; Schmidt et al., 2013). Furthermore, rats exhibit relatively large epididymal fat pads, which represent an ideal source for the isolation of sufficient amounts of MVF (Sato et al., 1987; Hoying et al., 1996).

In addition, it will be necessary to prove that the tissue within MVF/AV loop-containing chambers rapidly exhibits a functional blood perfusion, allowing its transfer to a defect site. For this purpose, several techniques have already been shown to be suitable. These include the intra-arterial injection of ink following chamber explantation (Bach et al., 2006; Kneser et al., 2006), microcomputed tomography (Arkudas et al., 2013) and sequential non-invasive magnetic resonance imaging (Hiscox et al., 2008).

Finally, the combination of MVF with AV loops should be evaluated in larger animal models, such as the pig or the sheep. These allow the creation of large-scale tissue defects that are comparable to those in humans (Morrison et al., 2016). However, before such studies can be conducted, the minimum number of MVF required to successfully pre-vascularize matrices used to surround AV-loops has yet to be determined. Once this parameter is evaluated, a rapid and sufficient vascularization of such particularly larger defects, which is of crucial importance for the successful translation of our suggested concept into clinical practice, may be possible.

## Conclusion

Particularly for clinical applications, the creation of an AV loop currently represents one of the most promising vascularization strategies in tissue engineering due to the opportunity of surgically anastomosing a pre-vascularized tissue construct with the blood vessels at a defect site. However, AV loop-based vascularization is a time-consuming process, which basically underlies the kinetics of sprouting angiogenesis. This problem may be overcome by the use of MVF. Their isolation and autologous transfer into AV loop-containing chambers may be feasible in an intra-operative one-step procedure and may markedly accelerate and improve the subsequent pre-vascularization of an AV loop-connected tissue construct, resulting in a significantly reduced hospitalization of patients. Hence, the herein introduced concept of combining two efficient vascularization strategies may pave the way for a broad application of AV loop-based tissue engineering in future clinical practice.

## Author Contributions

TS and ML drafted the manuscript. EA attended the conceptual discussion of the paper. MM performed a critical review of the manuscript. All authors contributed to the article and approved the submitted version.

## Conflict of Interest

The authors declare that the research was conducted in the absence of any commercial or financial relationships that could be construed as a potential conflict of interest.
